# Quantification of soya‐based feed ingredient entry from ASFV‐positive countries to the United States by ocean freight shipping and associated seaports

**DOI:** 10.1111/tbed.13881

**Published:** 2020-10-30

**Authors:** Gilbert Patterson, Megan C. Niederwerder, Gordon Spronk, Scott A. Dee

**Affiliations:** ^1^ One Health Solution VetNOW Canonsburg PA USA; ^2^ Department of Diagnostic Medicine/Pathobiology College of Veterinary Medicine Kansas State University Manhattan KS USA; ^3^ Pipestone Applied Research Pipestone Veterinary Services Pipestone MN USA

**Keywords:** ASFV, feed ingredients, seaports, soya bean meal, swine

## Abstract

African swine fever virus (ASFV) can survive in soya‐based products for 30 days with T ½ ranging from 9.6 to 12.9 days in soya bean meals and soya oil cake. As the United States imports soya‐based products from several ASFV‐positive countries, knowledge of the type and quantity of these specific imports, and their ports of entry (POE), is necessary information to manage risk. Using the data from the International Trade Commission Harmonized Tariff Schedule website in conjunction with pivot tables, we analysed imports across air, land and sea POE of soya‐based products from 43 ASFV‐positive countries to the United States during 2018 and 2019. In 2018, 104,366 metric tons (MT) of soya‐based products, specifically conventional and organic soya bean meal, soya beans, soya oil cake and soya oil were imported from these countries into the United States via seaports only. The two largest suppliers were China (52.7%, 55,034 MT) and the Ukraine (42.9%, 44,775 MT). In 2019, 73,331 MT entered the United States and 54.7% (40,143 MT) came from the Ukraine and 8.4% (6,182 MT) from China. Regarding POE, 80.9%–83.2% of soya‐based imports from China entered the United States at the seaports of San Francisco, CA, and Seattle, WA, while 89.4%–100% entered from the Ukraine via the seaports of New Orleans, LA, and Charlotte, NC. Analysis of five‐year trends (2015–2019) of the volume of soya imports from China indicated reduction over time (with a noticeably sharp decrease between 2018 and 2019), and seaport utilization was consistent. In contrast, volume remained high for Ukrainian soya imports, and seaport utilization was inconsistent. Overall, this exercise introduced a new approach to collect objective data on an important risk factor, providing researchers, government officials and industry stakeholders a means to objectively identify and quantify potential channels of foreign animal disease entry into the United States.

## 1 INTRODUCTION

As African swine fever virus (ASFV) continues to spread across Europe and Asia (Dixon et al., 2019), the United States Department of Agriculture has worked hard to identify potential risks for viral entry to the country and develop national response plans (USDA, 2020). While the primary focus has been on the risk of illegal entry of pork products, along with travellers from ASFV‐positive countries (Ito et al., 2020; Taylor et al., 2019), the possibility of ASFV entry via the importation of contaminated feed ingredients continues to gain recognition, based on a growing body of scientific evidence (Dee et al., 2018, 2020; Niederwerder et al., 2019; Stoian et al., 2019). Recent publications have described the transmission of ASFV to naïve pigs following consumption of contaminated feed, along with the calculation of the minimum infectious oral dose in feed (Niederwerder et al., 2019). Survival of ASFV in several feed ingredients has been documented out to at least 30 days post‐inoculation using shipping models simulating movement of feed ingredients from Eastern Europe to the United States (Dee et al., 2018; Stoian et al., 2019). A consistent observation across all these studies was the ability of ASFV to survive in soya‐based products, that is conventional (high protein/low fat) soya bean meal, organic (low protein/high fat) soya bean meal and soya oil cake, with reported half‐lives of 9.6, 12.9 and 12.4 days, respectively (Dee et al., 2018; Stoian et al., 2019). This information justifies the need to understand the countries of origin of these specific ingredients, the respective volumes imported and US ports of entry (POE) utilized. Access to these data would allow regulatory agencies to focus efforts and dedicate resources to a subset of critical ports, rather than the 329 US ports of entry (seaports, border crossings and airports) currently overseen by Customs and Border Protection (United States Customs & Border Protection, 2020). Therefore, the purpose of this short communication was to conduct an analytical exercise to generate this information.

## 2 METHODS

The exercise focused primarily on the years 2018 and 2019, but also evaluated data from 2015 to 2019. Information on the type and quantity of soya‐based feed ingredients and their specific POE was obtained at the International Trade Commission Harmonized Tariff Schedule website (www.hs.usitc.gov), a publicly available website that provides a transaction of specific trade commodities between the United States and its international trading partners. In the website database, each trade commodity was identified by a specific 10‐digit code known as the Harmonized Tariff Schedule (HTS), which was used for determining tariff classifications for all goods imported into the United States. Each commodity was classified based on the product's name, use and the material type, resulting in over 17,000 unique classification code numbers. Importing countries selected for inclusion in the analysis were obtained from the 43 ASFV‐positive countries listed on the Canadian Food Inspection Agency (CFIA) ASFV Watch List (Appendix A). These countries, spread across Asia, Africa and Europe, have been determined high‐risk areas for potential ASFV contamination of feed (Barr, 2019). Specific queries on eight specific HTS codes pertaining to soya‐based feed ingredients and the 43 countries were designed on the USITC website to create a comprehensive analysis which provided information on country of origin, quantity of product, year of entry and POE into the United States for each HTS code. Data were exported into Microsoft Excel and filtered into pivot tables to answer a series of questions:

1. What are the types of soya‐based products that enter the United States from the 43 ASFV‐positive countries?
2. Across the 43 ASFV‐positive countries, where do most of the soya‐based products come from?
3. What POE receive these high‐risk imports?
4. Do POE for soya‐based products change over time?

## 3 RESULTS

Upon completion of the analysis, answers to the questions were as follows:

QUESTION 1: WHAT ARE THE TYPES OF SOYA‐BASED PRODUCTS THAT ENTER THE UNITED STATES FROM THE 43 ASFV‐POSITIVE COUNTRIES?

The USITC database identified eight HTS codes that pertained to soya‐based feed ingredients: various types of soya beans, soya bean meal, soya oil cake and soya oil. These eight specific 10‐digit HTS codes were identified as soya‐based commodities with the potential to be included in swine diets (Table 1).

**TABLE 1:** Categorization of soya‐based commodities arriving at US POE

HTS Code	Description
1208.10.0000	FLOURS AND MEALS OF SOYBEANS, NESOI*
1201.90.0005	SOYBEAN OIL AND ITS FRACTIONS, FULLY REFINED, WASHED, BLEACHED OR DEODORIZED BUT NOT CHEMICALLY MODIFIED, NESOI
1201.90.0010	SOYBEAN OIL AND ITS FRACTIONS, ONCE‐REFINED (SUBJECT TO ALKALAI OR CAUSTIC WASH BUT NOT BLEACHED OR DEODORIZED), NOT CHEMICALLY MODIFIED
1201.90.0090	SOYBEAN OILCAKE AND OTHER SOLID RESIDUES RESULTING FROM THE EXTRACTION OF SOYBEAN OIL, WHETHER OR NOT GROUND OR IN THE FORM OF PELLETS
1208.10.0090	SOYBEAN SEEDS OF A KIND USED AS OIL STOCK, WHETHER OR NOT BROKEN
1507.90.4020	SOYBEAN SEEDS OF A KIND USED FOR SOWING
1507.90.4040	SOYBEANS, CERTIFIED ORGANIC, WHETHER OR NOT BROKEN, EXCEPT SEEDS OF A KIND USED FOR SOWING OR USED AS OIL STOCK
2304.00.0000	SOYBEANS, WHETHER OR NOT BROKEN, OTHER THAN CERTIFIED ORGANIC, NESOI

Abbreviations: HTS, Harmonized Tariff Schedule; POE, ports of entry.

* NESOI: Refers to ‘Not Elsewhere Specified or Indicated’.

QUESTION 2: ACROSS THE 43 ASFV‐POSITIVE COUNTRIES, WHERE DO MOST OF THE SOYA‐BASED PRODUCTS COME FROM?

The next step was to identify the country of origin and the volume of soya‐based products that entered the United States. The analysis indicated that in 2018 the United States imported a total of 104,707 metric tons (MT) of the eight previously identified commodities from eight of the 43 countries on the CFIA watch list: China, Ukraine, Russia, Uganda, Belgium, Togo, Vietnam and Thailand (Table 2). Of this total volume, 55,101 MT (52.6%) of these designated soya‐based ingredients were imported from China. Ukraine was the second largest exporter of soya‐based products into the United States with 44,776 MT (42.8%) (Table 2). In contrast, during 2019 the United States imported a total of 73,331 metric tons (MT) of soya‐based products with 40,143 MT (54.7%) imported from the Ukraine and 6,182 *M* (6.8%) from China.

**TABLE 2:** Total volume and country of origin of soya‐based imports in 2018–2019

Country of Origin	Sum of 2018 (MT)	% of Total 2018	Sum of 2019 (MT)	% of Total 2019
Ukraine	44,776	42.9%	40,143	54.7%
Russia	3,396	3.3%	20,661	28.2%
China	55,039	52.7%	6,182	8.4%
Moldova	0	0.0%	5,986	8.2%
Belgium	143	0.1%	244	0.3%
Togo	22	0.0%	113	0.2%
Vietnam	0	0.0%	1	0.0%
Uganda	990	0.9%	0	0.0%
Grand Total	104,366	100.0%	73,331	100.0%

Based on these data, ingredient profiles of China and the Ukraine imports were developed. The primary ingredients imported from China in 2018 were ground or pelletized soya oil cake (41,998 MT, 76.2%) and organic soya beans (7,780 MT, 14.1%), while the primary product imported from the Ukraine was organic soya beans at 44,776 MT (99.9%). Similar results were seen in data from 2019 with 4,449 MT (72.9%) of soya oil cake and 1,482 MT (23.9%) of organic soya beans imported from China and 40,143 MT (100%) of organic soya beans arriving from the Ukraine (Table 3).

**TABLE 3:** Volume analysis of individual soya‐based ingredients from China and the Ukraine to the United States in 2018 and 2019

China and Ukraine Compared in 2018–2019	Sum of 2018 (MT)	Sum of 2019 (MT)
*CHINA*	*55,039*	*6,182*
SOYBEAN OILCAKE AND OTHER SOLID RESIDUES RESULTING FROM THE EXTRACTION OF SOYBEAN OIL, WHETHER OR NOT GROUND OR IN THE FORM OF PELLETS	41,998	4,449
SOYBEANS, CERTIFIED ORGANIC, WHETHER OR NOT BROKEN, EXCEPT SEEDS OF A KIND USED FOR SOWING OR USED AS OIL STOCK	7,780	137
SOYBEANS, WHETHER OR NOT BROKEN, OTHER THAN CERTIFIED ORGANIC, NESOI	5,112	1,482
SOYBEAN OIL AND ITS FRACTIONS, ONCE‐REFINED (SUBJECT TO ALKALAI OR CAUSTIC WASH BUT NOT BLEACHED OR DEODORIZED), NOT CHEMICALLY MODIFIED	103	0
FLOURS AND MEALS OF SOYBEANS, NESOI	21	78
SOYBEAN SEEDS OF A KIND USED AS OIL STOCK, WHETHER OR NOT BROKEN	18	30
SOYBEAN OIL AND ITS FRACTIONS, FULLY REFINED, WASHED, BLEACHED OR DEODORIZED BUT NOT CHEMICALLY MODIFIED, NESOI	7	7
*UKRAINE*	*44,776*	*40,143*
SOYBEANS, CERTIFIED ORGANIC, WHETHER OR NOT BROKEN, EXCEPT SEEDS OF A KIND USED FOR SOWING OR USED AS OIL STOCK	44,775	40,143
SOYBEAN OIL AND ITS FRACTIONS, FULLY REFINED, WASHED, BLEACHED OR DEODORIZED BUT NOT CHEMICALLY MODIFIED, NESOI	1	0
SOYBEANS, WHETHER OR NOT BROKEN, OTHER THAN CERTIFIED ORGANIC, NESOI	0	0
SOYBEAN SEEDS OF A KIND USED AS OIL STOCK, WHETHER OR NOT BROKEN	0	0
Grand Total	99,814	46,325

QUESTION 3: WHAT POE RECEIVE THESE HIGH‐RISK IMPORTS?

The next step was to identify the US seaports that received soya‐based imports from the 43 ASFV‐positive countries in 2018 and 2019. Thirty‐seven seaports imported 177,697 MT of soya‐based imports over this period, with New Orleans, Charlotte, NC, San Francisco, CA, and Seattle, WA, accounting for 88.6% of imports (157,574 MT) (Table 4a). Based on data from question 2, POE summaries specific for China (Table 4b) and the Ukraine (Table 4c) were conducted. In 2018, a total of four POE received greater than 88% of all of soya‐based imports from China: San Francisco/Oakland, CA (60.36%), Seattle, WA (20.54%), Baltimore, MD (4.13%), and Los Angeles, CA (3.78%). In 2019, 70.4% of soya‐based imports entered the port of San Francisco, 12.8% entered the port of Seattle, while 0.8 and 7.3% entered at the ports of Baltimore and Los Angeles, respectively (Table 4b). Regarding the Ukraine, in 2018 and 2019 most soya‐based products entered the United States via the seaports of New Orleans, LA, and Charlotte, NC, with a small amount entering via the port of Baltimore in 2018 (Table 4c).

**TABLE 4A:** Ports of entry (POE) summary across all countries surveyed during 2018–2019

POE	Sum of 2018 (MT)	Sum of 2019 (MT)
New Orleans, LA	36,268	47,065
Charlotte, NC	5,261	18,925
San Francisco, CA	33,261	4,469
Ogdensburg, NY	3,166	801
Seattle, WA	11,532	793
Los Angeles, CA	2,085	454
New York, NY	418	352
Cleveland, OH	143	181
Chicago, IL	1,553	112
Norfolk, VA	1,526	64
Baltimore, MD	7,995	51
St. Louis, MO	0	33
Savannah, GA	18	30
Houston‐Galveston, TX	637	1
Mobile, AL	0	1
Tampa, FL	0	0
Honolulu, HI	0	0
San Juan, PR	0	0
Buffalo, NY	0	0
Boston, MA	0	0
Great Falls, MT	60	0
Columbia‐Snake, OR	433	0
St. Albans, VT	0	0
Philadelphia, PA	0	0
Detroit, MI	0	0
Miami, FL	0	0
Minneapolis, MN	11	0
Grand Total	104,366	73,331

**TABLE 4B:** Ports of entry (POE) summary: China during 2018–2019

POE	Sum of 2018 (MT)	Sum of 2019 (MT)
San Francisco, CA	33,261	4,355
Seattle, WA	11,302	793
Baltimore, MD	2,275	51
Los Angeles, CA	2,085	454
Chicago, IL	1,531	112
Norfolk, VA	1,526	1
New Orleans, LA	1,484	0
Houston‐Galveston, TX	637	1
Columbia‐Snake, OR	433	0
New York, NY	417	352
Great Falls, MT	60	0
Savannah, GA	18	30
Minneapolis, MN	11	0
Honolulu, HI	0	0
Cleveland, OH	0	0
Tampa, FL	0	0
Detroit, MI	0	0
St. Louis, MO	0	33
Boston, MA	0	0
Buffalo, NY	0	0
Mobile, AL	0	1
Grand Total	55,039	6,182

**TABLE 4C:** Ports of entry (POE) summary: Ukraine during 2018–2019

POE	Sum of 2018 (MT)	Sum of 2019 (MT)
New Orleans, LA	34,784	26,218
Charlotte, NC	5,261	13,925
Baltimore, MD	4,729	0
Grand Total	44,775	40,143

QUESTION 4: DO POE FOR SOYA‐BASED PRODUCTS CHANGE OVER TIME?

**FIGURE 1:**
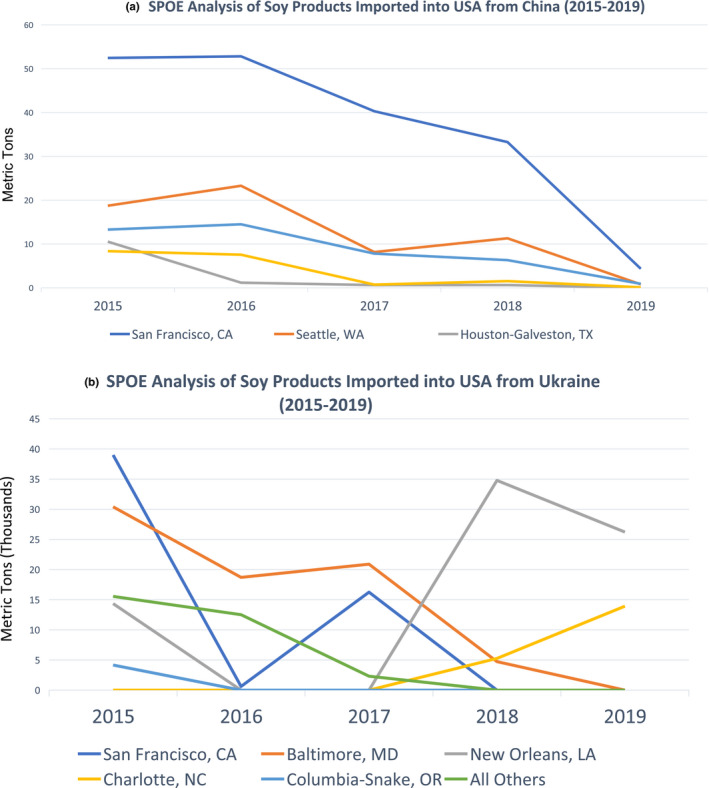
(a) Ports of entry analysis of soya products imported into the United States from China (2015–2019). (b) Ports of entry analysis of soya products imported into the United States from Ukraine (2015–2019) [Colour figure can be viewed at wileyonlinelibrary.com]

The final segment of the analysis focused on whether the volume of soya‐based imports and POE changed over time, once again focusing on China (Figure 1a) and the Ukraine (Figure 1b). To evaluate trends over time, data were evaluated from 2015 to 2019. Different patterns between countries were observed, with imports from China demonstrating a reduction in volume over time with consistent POE, while data from the Ukraine indicated variability across POE, while the overall volume imported to the United States remained high.

## 4 DISCUSSION

As the US feed supply becomes increasingly globalized, the risk of foreign animal diseases entering the country is significantly increased, particularly when dealing with agricultural trade commodities from countries endemically infected with foreign animal diseases. Although expanding international trade allows access to diverse and competitive trade markets, the loss in direct oversight reduces commodity quality control and safety. Therefore, based on the growing body of evidence regarding the ability of foreign animal disease pathogens such as ASFV to survive in feed, it is imperative that swine feed ingredients imported into the United States from endemically infected countries be treated with increased scrutiny and caution (Patterson et al., 2019). Obviously, this presents an immeasurable challenge for US CBP due to the sheer volume of imported products and the vast number of seaports in the United States. In the absence of unlimited resources, it is important to focus on areas where the risk of disease entry is the highest. In response to this challenge, we conducted this analysis to provide information on the importation of high‐risk ingredients from ASFV‐positive countries and the corresponding POE. Based on volume of imports, we focused on a country from Asia (China) and one from Eastern Europe (the Ukraine), identified where soya‐based products entered the United States and evaluated change in volume and seaport utilization over time. Regarding China, it was interesting to see the consistency of POE utilization and how imports of soya‐based products decreased, particularly from 2018 to 2019. When seeking an explanation for this change, the US soya industry reported that drivers of change were peer‐reviewed publications demonstrating the survival of viruses in soya‐based products, the resulting swine industry‐driven trade press sharing this information and the response from producer stakeholders (P. Lobo, personal communication, July 10, 2020). Of significant impact was a letter written by the National Pork Producers Council to the US Secretary of Agriculture, signed by all major pork‐producing states, requested assistance via the Animal Health Protection Act to prohibit soya‐based imports from China (NPPC, 2020). In contrast, seaport utilization involving Ukraine imports was inconsistent and volumes imported remained high, which showcased the ability of our approach to identify new areas of risk which had previously gone unnoticed.

Despite these strengths, this approach was not without limitations. Table 1 describes eight specific 10‐digit HTS codes that were selected to be included in this study based on their potential to both harbour viable virus and be fed to pigs; however, each of these products does not share the same amount of risk to the US swine population. For example, of these eight specific products, only soya beans, soya oil cake and soya bean meal are significant risks in terms of both their likelihood to be fed to pigs and their documented ability to enhance survival of ASFV for extended periods. These products were also deemed high risk because they are major components of swine rations throughout the industry. Another limitation of the approach was the lack of information on final product destination or intended use; therefore, it was not possible to determine how much of a product ultimately ends up in the domestic swine supply chain. In addition, the numbers presented in this study indicated the total volume of a specific product cleared by US Customs at POE. USITC defines these products as ‘imports for consumption’, intended for use and distribution across all industries and markets and did not provide any further information on final product destination or intended use; therefore, our methods could not determine how much of a product ultimately ends up in the domestic swine supply chain. Furthermore, given the enormous interconnected web that is the modern global trade network, there remains some speculation of the true origin of trade products as they arrive on US shores. For example, countries may import products from one country, only to repackage them and export to another. Therefore, these data are limited to only the immediate importing country and it is not capable to determine complete travel histories of all products that clear US Customs.

In closing, we felt that the exercise was successful and enhanced the knowledge of the topic. We set out to answer four specific questions using a novel approach which gathered information that is important for the development of science‐based feed biosecurity plans. While we focused on soya‐based products and ASFV‐positive countries, this same approach could be applied to multiple foreign trade commodities, which could assist in the development of both human and animal food safety protocols. It is hoped that these efforts will continue to stimulate communication and collaboration between the feed and livestock industries, resulting in further research into the emerging concept of ‘global feed biosecurity’. Ideally, current and future information regarding the risk of pathogen spread in feed will enhance the accuracy of risk assessments, drive the continual development of efficacious feed‐based mitigation strategies and ultimately bring the health status in the country of origin into the forefront of philosophies regarding the global trade of feed ingredients.

## CONFLICT OF INTEREST

The authors report no conflicts of interest.

## ETHICAL APPROVAL

No animals were used in this project.

## Data Availability

All data from the study were made available in the paper.

## Appendix A

### CFIA WATCH LIST COUNTRIES

**Table:**

Belgium	Ghana	Romania
Benin	Guinea‐Bissau	Russia
Burkina Faso	Hungary	Rwanda
Bulgaria	Italy	Senegal
Burundi	Kenya	Sierra Leone
Cabo Verde	Latvia	South Africa
Cameroon	Lithuania	Tanzania
Central African Republic	Madagascar	Togo
Chad	Malaw	Uganda
China	Moldova	Ukraine
Congo	Mongolia	Vietnam
Cote D'Ivoire	Mozambique	Zambia
Czech Republic	Namibia	Zimbabwe
Estonia	Nigeria	
Gambia	Poland	
